# A Single-Cell Approach to the Elusive Latent Human Cytomegalovirus Transcriptome

**DOI:** 10.1128/mBio.01001-18

**Published:** 2018-06-12

**Authors:** Felicia Goodrum, Shannon McWeeney

**Affiliations:** a University of Arizona, Tucson, Arizona, USA; b Oregon Health and Sciences University, Portland, Oregon, USA

**Keywords:** MARS-Seq, cytomegalovirus, herpesviruses, scRNA-seq, transcriptome

## Abstract

Herpesvirus latency has been difficult to understand molecularly due to low levels of viral genomes and gene expression. In the case of the betaherpesvirus human cytomegalovirus (HCMV), this is further complicated by the heterogeneity inherent to hematopoietic subpopulations harboring genomes and, as a consequence, the various patterns of infection that simultaneously exist in a host, ranging from latent to lytic. Single-cell RNA sequencing (scRNA-seq) provides tremendous potential in measuring the gene expression profiles of heterogeneous cell populations for a wide range of applications, including in studies of cancer, immunology, and infectious disease. A recent study by Shnayder et al. (mBio 9:e00013-18, 2018, https://doi.org/10.1128/mBio.00013-18) utilized scRNA-seq to define transcriptomal characteristics of HCMV latency. They conclude that latency-associated gene expression is similar to the late lytic viral program but at lower levels of expression. The study highlights the numerous challenges, from the definition of latency to the analysis of scRNA-seq, that exist in defining a latent transcriptome.

## COMMENTARY

Herpesviruses are incurable due to their ability to establish a latent infection that cannot be targeted by current antivirals. The persistence of herpesviruses through latency results in an enduring health risk, as their reactivation from latency is associated with significant disease, particularly in immunocompromised individuals. Understanding viral latency at the molecular level offers the opportunity to develop strategies to identify and target latently infected cells and to reveal mechanisms of viral persistence.

Viral latency, as classically defined, is the maintenance of viral genomes in the absence of progeny virus production, but with the ability to reactivate for subsequent rounds of replication. During latency, the viral genome is chromatinized, and the latent transcriptional program has been presumed to be restricted to a limited number of viral RNAs, proteins, and microRNAs. Indeed, latency has often been defined experimentally as the absence of a single lytic gene in the presence of a single latency-associated gene. However, highly sensitive, global analysis of gene expression during infection with human cytomegalovirus (HCMV) in cell types supporting a latent infection paints a far more complex picture of viral gene expression associated with latent states (1–5). Similar findings have been made for alphaherpesviruses (6–10). Therefore, Shnayder et al.’s study (11) joins a growing body of herpesvirus literature that challenges the paradigm that latency is a transcriptionally silent state, but with the surprising conclusion that there is no unique latent transcriptome, suggesting a model whereby latency reflects a quantitative, not qualitative, difference in viral gene expression from that of the replicative state.

Two major challenges to defining the latent transcriptome of HCMV exist. First, the viral genome is estimated to be maintained at 2 to 13 copies per cell in ≤1 in 10,000 mononuclear cells in an infected host (12), and transcription is restricted (13, 14). Second, HCMV establishes latency in hematopoietic progenitor cells (HPCs), which are a heterogeneous population of cells with dynamic programs of differentiation that are sensitive to *ex vivo* stimulation. This heterogeneity especially complicates transcriptome analysis, since HCMV reactivation from latency is associated with myeloid lineage differentiation (15, 16). Therefore, it follows that the viral transcriptome may vary along a spectrum from latent to replicative in cells at different stages of differentiation within an HPC population.

To define viral genes associated with natural infection in the host, Shnayder et al. searched the existing bank of transcriptome sequencing (RNA-seq) data for the presence of HCMV transcripts across human tissues (11). The paucity of HCMV gene expression in seropositive (presumably latently infected) individuals is reflected in the fact that an analysis of 1.5 billion aligned sequence reads from 25 samples of hematopoietic origin identified no bona fide CMV reads. Further analysis of the GTEx database (433 billion reads derived from 9,416 RNA-seq samples from 549 individuals spanning 31 tissues) identified only 6 individuals with ≥10 reads in any tissue. Hierarchical clustering of samples with >4 reads resolved two patterns of gene expression, one dominated by long noncoding RNAs (lncRNAs) (group I) and another dominated by immediate early genes (group II). Group I and II correlated to infection in fibroblasts at late times (24 to 72 h postinfection [hpi]) and early times (3 to 5 hpi), respectively. However, caution must be taken with these correlations given the small number of genes detected and the low abundance of reads. Further, because lncRNAs are the most abundantly expressed transcripts in any context of infection (2, 17–19), the presence of lncRNAs should not be taken as evidence of a lytic infection. One approach to the challenge of low-abundance HCMV reads in seropositive individuals is the use of targeted enrichment approaches to increase the proportion of viral reads in a mixed transcriptome (1).

Single-cell RNA sequencing (scRNA-seq) following *in vitro* infection has the potential to skirt the issues imposed by heterogeneity of the cell population and, indeed, offers a powerful approach to defining the heterogeneity of the viral transcriptome across the hematopoietic hierarchy of differentiation. In the study by Shnayder et al., 3,655 CD14^+^ cells were analyzed by massively parallel RNA single-cell sequencing framework (MARS-Seq) analysis and formed 6 clusters based on host and viral gene expression. In clusters 1 to 6, viral gene expression ranged from >10% to 0% of the entire transcriptome, respectively (11). Due to its high proportion of viral gene expression, cluster 1 was concluded to represent lytically infected cells. This conclusion is based, in part, on the correlation to the HCMV transcriptome in monocyte-derived macrophages and fibroblasts. The high correlation between the cluster 1 CD14^+^ transcriptome and the transcriptome from infection in fibroblasts and macrophages is likely driven by the high expression of lncRNAs that mask the impact of the large number of genes that were expressed in fibroblasts or macrophages but not expressed in CD14^+^ monocytes across the different clusters. Additional sensitivity analysis, with the highly abundant lncRNAs removed, to assess the conclusions of both the clustering and the correlation would be important to confidently compare these infection models. Further, it is interesting to note that essentially all cells represented in cluster 1 were from the early time points of infection (3, 4, and 5 days postinfection [dpi]). It is expected that lytically infected cells with this profile of gene expression in the population would be represented in all of the time points and, perhaps to an even greater extent, at later time points postinfection. Because cluster 1 is comprised of cells at early time points postinfection, it is possible that these cells undergo an initial burst of gene expression prior to establishing a more quiescent state and might influence latency, as described for alphaherpesviruses (6, 20–22).

The CD14^+^ gene expression clusters continued to segregate by time postinfection, with clusters 5 and 6 being comprised predominantly of cells from 7 and 14 dpi, respectively. The latter clusters are comprised of cells with little to no evident viral gene expression (<0.1% of total reads were HCMV reads). In cells where no viral transcripts were detected, the viral genome may be highly repressed or these cells may not have been initially infected, since the frequency of infections based on green fluorescent protein expression from the viral genome was ~62%. Further, due to the cutoff of ≥1,000 reads per cell, it is possible that the depth of sequencing may not be able to capture the full transcriptome, particularly for very-low-abundance RNAs. Measuring the maintenance of viral genomes over this time course is essential to understanding whether this apparent loss of HCMV gene expression over time is due to the establishment of a more quiescent but reactivatable state or whether it reflects an abortive infection where viral genomes are lost.

scRNA-seq analysis of infected CD34^+^ HPCs presented even greater challenges than CD14^+^ cells due to the yet-lower abundance of viral transcripts and to the fact that transcripts were detected in only ~5% of the cells sequenced. Again, it is difficult to ascertain whether the failure to detect transcripts is due to their low abundance or to a truly quiescent infection or because the cells were not infected. Shnayder et al. compared the HCMV transcriptome in CD34^+^ HPCs to that in cluster 1 (>10% viral gene expression) from CD14^+^ monocytes and concluded that the CD34^+^ HPC latency-associated transcriptome largely mirrored that of a lytic infection, being differentiated from a lytic state only by the level of expression (11). The associated correlation (Spearman coefficient of 0.67) is likely heavily driven by high expression of lncRNAs in both contexts, despite the fact that a large number of genes are expressed in a “lytic” infection but not in CD34^+^ HPCs. Further, because CD34^+^ scRNA-seq was performed at 4 dpi, latency may not yet have been established and the gene expression observed may reflect an early burst of gene expression.

While these studies reveal a transcriptome associated with infection in hematopoietic subpopulations that extends beyond a few genes, consistent with the results of a number of contemporary studies (1, 2), the conclusion that there is no difference in latent and lytic transcriptomes is more difficult to assert. A critical caveat in interpreting scRNA-seq analysis is the sparsity of the data, i.e., the high proportion of zero read counts (23, 24), which often can be due to technical reasons (such as “dropouts,” where a gene is expressed but not detected in a sequencing run) rather than the biological factors or interest (lack of expression in a specific cell type or time point). The limitation in scRNA-seq coverage requires ≥50,000 reads for unbiased transcriptome definition from low-abundance transcripts (25) and represents a remaining challenge to defining the latent HCMV transcriptome in cells with low levels of viral gene expression. Given this, as well as the inherently stochastic and heterogeneous nature of scRNA-seq, care must be taken with regard to appropriate normalization to address within-sample (e.g., GC content bias, transcript length) and between-sample (e.g., sequencing depth) issues. The unique molecular identifier (UMI)-based protocol utilized by Shnayder et al. can remove sequencing depth bias if the libraries are sequenced to saturation, but UMIs still cannot account for capture efficiency or cellular mRNA content differences (26). While the authors estimated leakage noise in the MARS-seq analysis, no formal normalization was conducted to address these technical issues, and only raw reads were utilized for the 10× sequencing data. Further, while scRNA-seq offers immense promise in understanding the variation in profiles of gene expression between cells in a population, challenges in defining complex transcriptomes, such as those comprised of viral and host sequences, remain.

The study by Shnayder et al. brings the power of single-cell sequencing to HCMV infection and demonstrates an array of profiles of viral mRNAs and lncRNAs, from broad to narrow, expressed in CD14^+^ and CD34^+^ hematopoietic cells infected *in vitro* (11). Going forward, it will be important to understand the profiles of gene expression associated with specific cell types within the larger CD14^+^ and CD34^+^ populations and how both viral and host gene expression changes over time and cellular differentiation from initial infection to the establishment of latency and, finally, reactivation.
